# Clinical Outcomes and Magnetic Resonance Imaging Evaluation of Autologous Protein Solution Treatment for Knee Osteoarthritis

**DOI:** 10.7759/cureus.84698

**Published:** 2025-05-23

**Authors:** Chi Fangzhou, Tomoharu Mochizuki, Takashi Ushiki, Hiroyuki Kawashima

**Affiliations:** 1 Division of Orthopedic Surgery, Niigata University Medical and Dental Hospital, Niigata, JPN; 2 Department of Transfusion Medicine, Cell Therapy and Regenerative Medicine, Niigata University Medical and Dental Hospital, Niigata, JPN

**Keywords:** aps, clinical efficacy, k-l grade, knee osteoarthritis, retrospective analysis

## Abstract

Objective: This study aims to investigate the clinical outcomes and magnetic resonance imaging evaluation (MRI) of intra-articular injection of autologous protein solution (APS) in patients with knee osteoarthritis of varying severities.

Methods: A retrospective analysis was conducted on the clinical data of consecutive subjects with knee osteoarthritis (KOA) of varying Kellgren-Lawrence (KL) grades who underwent a single APS injection. The Knee injury and Osteoarthritis Outcome Score (KOOS), covering symptoms (S), pain (P), activity (A), sports (SP), and quality of life (Q), was used to evaluate the patients at pre-treatment and 12 months post-treatment. Minimal Clinically Important Difference (MCID) and Osteoarthritis Research Society International Set Responder Criteria Osteoarthritis Clinical Trials Revisited (OMERACT-OARSI) tools were used to observe improvement in different KL grades. Additionally, an MRI was performed pre-treatment and 12 months post-treatment. Semi-quantitative analysis (Magnetic Resonance Imaging Osteoarthritis Knee Score (MOAKS)) was applied to assess bone marrow lesions (BMLs), articular cartilage, osteophytes, synovitis and effusion, and meniscal lesions in various subregions of the knee joint.

Results: A total of 53 patients (66 knees) were included in the final analysis. At 12 months post-treatment, overall KOOS scores showed improvement. The responder rates were 78.6% (11 knees) in KL2, 68.4% (13 knees) in KL3, and 51.5% (17 knees) in KL4. Significant improvements were observed in KOOS-S, KOOS-P, and KOOS-Q across all patients. KOOS-A and KOOS-SP demonstrated statistically significant differences only in the KL2 and KL4 groups (P < 0.05). Comparisons of KOOS score differences between groups revealed that patients in the KL2 group experienced greater improvements in activity levels and quality of life compared to those in the KL3 and KL4 groups (P < 0.05). MOAKS evaluation revealed no significant improvement in cartilage damage, BMLs, synovitis-effusion, and meniscal status. In the KL4 group, the post-treatment scores for patellar superior and inferior osteophytes were higher than the pre-treatment scores (P = 0.039).

Conclusion: This study provides evidence supporting the clinical efficacy of a single intra-articular injection of APS in KOA. However, the therapeutic effect of APS for structural changes in imaging remains limited.

## Introduction

As a multifactorial chronic disease, osteoarthritis (OA) has a high incidence worldwide [1]. The knee joint, as a common site of OA, is characterized by progressive cartilage degeneration, narrowing the joint space until it is lost [2]. With the aging of the population, the incidence of knee OA (KOA) continues to rise annually [3]. For the treatment of KOA, a stepwise treatment strategy has been used in clinical practice. A stepwise treatment strategy is used to achieve a certain degree of alleviated pain and improved joint function through systematic conservative treatment; while some patients respond well to the existing non-surgical treatments and have good outcomes without surgery, others experience progressive symptoms worsening over several years and eventually require surgical intervention [4]. Therefore, it is essential to explore broadly effective conservative treatment options.

Platelet-rich plasma (PRP) is used as a product containing a high concentration of platelets obtained by centrifugation of one's whole blood, which contains three to five times more platelets than whole blood cells. PRP has emerged as a promising conservative treatment option due to the release of bioactive components (e.g., platelet-derived growth factor (PDGF), transforming growth factor-β (TGF-β), and vascular endothelial growth factor (VEGF)) upon platelet activation, which is hypothesized to play a positive role in the repair of tissue damage [5]. Similarly, studies over the past decade have demonstrated the efficacy of PRP in alleviating symptoms in patients with early-stage OA, showing significantly better outcomes compared to traditional conservative treatments [6].

Autologous protein solution (APS) is a derivative of autologous peripheral blood-derived orthopedic biologics (APBO) of PRP. It is prepared using the nSTRIDE APS Kit (Zimmer Biomet Holdings, Inc., Warsaw, Indiana, United States). This process produces a product with high leukocyte counts and high levels of anti-inflammatory mediators, including interleukin-1 receptor antagonist (IL-1Ra), soluble TNF receptor II (sTNF-RII), and a high IL-1Ra/IL-1β ratio. These characteristics make APS different from the more commonly used leukocyte-poor PRP in both preparation method and cell composition. APS is classified as a novel leukocyte-rich PRP formulation [7-9]. However, there are few studies on its clinical efficacy and mechanism as a novel autologous therapy for the treatment of osteoarthritis of the knee [10,11]. Therefore, more observational studies on the clinical therapeutic effects of APS have become particularly important and can provide supporting evidence for clinical application.

MRI offers superior visualization of soft tissue and cartilage morphology compared to plain radiographs. The MRI Osteoarthritis Knee Score (MOAKS) is a validated semiquantitative tool that systematically evaluates key structural features, cartilage integrity, bone marrow lesions, osteophytes, synovitis-effusion, and meniscal status, to detect early or subtle changes in joint pathology [12]. We adopted MOAKS in this study to obtain a more reliable measure of structural progression in KOA.

While previous studies on the therapeutic effect of APS have mostly used clinical scores as the main assessment index [13,14], the relationship between symptomatic relief and underlying structural remodeling remains unclear. By combining KOOS with MRI-based MOAKS assessments, our study addresses this gap, providing simultaneous evaluation of both clinical and structural responses to APS injections in KOA patients across varying Kellgren-Lawrence grades. Since APS is an alternative treatment method, it is important to apply it to patient groups where it can achieve the best results. This study aims to observe the effects of APS in patients with KOA of different severities and explore the variability and effectiveness. Additionally, MRI findings, such as synovitis, joint effusion, and bone marrow lesions (BMLs), are closely related to the progression of KOA [15]. Therefore, this study also uses MRI to evaluate treatment outcomes, aiming for a more thorough assessment of its efficacy on KOA with different severities.

## Materials and methods

The study was conducted according to the ethical principles suggested in the Declaration of Helsinki and was approved by the institutional review board of Niigata University Medical and Dental Hospital, Niigata, Japan (IRB number: 2022-0218). All the participants provided informed consent before participating in the survey.

Patients

Retrospective data were collected from patients with KOA (Kellgren-Lawrence (KL) grade of 1-4 (KL1-4)) who received a single intra-articular APS injection in the orthopedics department of our hospital between 2020 and 2023. All evaluations were performed by experienced orthopedic surgeons according to standardized criteria to reduce inter-observer variability. The Kellgren-Lawrence (KL) grading system is used to classify the severity of osteoarthritis based on X-ray findings [16]. Grade 1 indicates very early or doubtful changes, with possible small bone spurs but no clear joint space narrowing. Grade 2 represents mild osteoarthritis, where definite osteophytes are visible, and joint space narrowing may begin. Grade 3 is considered moderate OA, with multiple osteophytes, clear joint space narrowing, and signs of bone sclerosis. Finally, Grade 4 is severe osteoarthritis, showing large osteophytes, significant loss of joint space, bone hardening, and deformity. Patients were screened according to the following criteria.

Patients were eligible for inclusion if they met the following criteria: (1) complete treatment records, including knee magnetic resonance imaging (MRI) scans and Knee injury and Osteoarthritis Outcome Score (KOOS) data from both the initial visit and follow-up; (2) age over 18 years; (3) presence of long-term limitations in daily living activities; and (4) a diagnosis of KOA based on established clinical criteria, with a baseline Kellgren-Lawrence (KL) grade of 1-4, including those with coexisting meniscal lesions or synovitis on baseline imaging. Exclusion criteria were as follows: (1) known allergy to any components of the treatment; (2) receipt of intra-articular corticosteroid or hyaluronic acid injections within the preceding two weeks; (3) presence of systemic inflammatory conditions (e.g., rheumatoid arthritis), malignancies, hematological disorders, infections, immunodeficiencies, or other metabolic diseases; (4) diagnosis of psychiatric disorders; (5) severe joint diseases, such as septic arthritis, gout, or ankylosing spondylitis; and (6) recent use of anticancer or immunosuppressive medications. A total of 55 patients (68 knees) received APS treatment. Initial and follow-up knee joint scores, as well as imaging evaluations, were conducted by experienced orthopedic specialists. All patients completed follow-up 12 months after treatment. All patients who received treatment did not undergo any other treatments during the one-year follow-up period.

APS preparation and application

Preparation of APS

APS is prepared using the nSTRIDE APS Kit (Zimmer Biomet, Warsaw, IN, USA). In the first step, 55 mL of blood and 4 mL of anticoagulant citrate dextrose solution A (Citra Labs, Braintree, MA, USA) are injected into the nSTRIDE Cell Separator. After centrifugation at 3,200 rpm for 15 minutes, approximately 6 mL of platelet-rich plasma (PRP) is obtained. The PRP is then transferred to the next step, the nSTRIDE Concentrator, where it is exposed to polyacrylamide beads and filtered through centrifugation at 2,000 rpm for two minutes, producing approximately 2-3 mL of APS.

Intra-articular Injection of APS

Within 30 minutes of preparation, APS was injected into the knee joint under ultrasound guidance using a 23-gauge needle. Following joint puncture, as much joint fluid as possible was aspirated before the APS injection. After the injection, patients were instructed to rest at home on the day of the procedure. If knee swelling or pain occurred after the injection, they were advised to cool the affected area to relieve pain. If the pain persisted, one acetaminophen tablet was allowed as rescue medication. From the day following the procedure, no activity restrictions were applied, and patients were permitted to gradually resume sports and recreational activities according to their pain tolerance.

Evaluation

Pre-treatment and at 12 months post-treatment, patients were clinically evaluated using KOOS to assess outcomes, determining the primary clinical effects of APS therapy. MRI was performed at baseline and 12 months post-treatment. All MRI scans were acquired using a 3.0-Tesla scanner (Discovery MR 750w; GE Healthcare, Milwaukee, USA). Coronal and sagittal T2-weighted images were obtained with a repetition time of 4,025-4,500 ms, an echo time of 100 ms, a slice thickness of 3.0-4.0 mm with 1.0 mm spacing, a field of view of 180 mm, and a matrix size of 448×256. Coronal and sagittal T1-weighted images were obtained with a repetition time of 450-500 ms, an echo time of 7-8 ms, and the same slice thickness, spacing, field of view, and matrix size. The actual time interval from APS injection to the follow-up MRI scan was 12.27 ± 1.20 months (mean ± SD). Demographic and clinical characteristics, including age, pre-treatment range of motion, OA severity based on KL grading, and body mass index (BMI), were recorded. Clinical assessments included the KOOS, covering symptoms (S), pain (P), activity (A), sports (SP), and quality of life (Q), with higher scores indicating better knee function [17]. The Minimal Clinically Important Difference (MCID) was determined using a distribution-based approach, specifically the Half Standard Deviation (0.5 × SD) method. MCID was defined as half of the standard deviation of the baseline KOOS scores, based on the assumption that a change equivalent to 0.5 standard deviations reflects a statistically meaningful clinical improvement [18]. The Osteoarthritis Research Society International Set Responder Criteria Osteoarthritis Clinical Trials Revisited (OMERACT-OARSI) set of responder criteria was used to calculate the number of responders/non-responders to APS treatment over time [19] (Appendix A). Additionally, MRI findings were evaluated using the Semi-quantitative analysis (Magnetic Resonance Imaging Osteoarthritis Knee Score (MOAKS)) to assess BMLs and cysts, articular cartilage, osteophytes, Hoffa’s synovitis, synovial effusion, and meniscus [12]. Details are described in Appendix B.

To evaluate scoring reproducibility, a random of 10 knee MRIs was re-scored by the same orthopedic surgeon (intra-rater assessment) and independently by a second orthopedic surgeon (inter-rater assessment). The concordance rates for KL grading were 0.89 (intra-rater) and 0.86 (inter-rater); for the MOAKS cartilage damage score, concordance rates were 0.75 (intra-rater) and 0.66 (inter-rater).

Statistical analysis

The Shapiro-Wilk test was used to assess the normality of the data. As the data did not follow a normal distribution, median and interquartile range (IQR) were used for descriptive statistics. Within-group comparisons between pre-treatment and one-year post-treatment data were conducted using the Wilcoxon signed-rank test, while between-group comparisons of pre- and post-treatment differences were performed using the Kruskal-Wallis test. Post hoc analyses with Bonferroni correction were applied for parameters showing statistically significant differences. Statistical significance was set at P < 0.05. All statistical analyses were performed using SAS 9.4 statistical software (SAS Institute Inc., Cary, North Carolina, United States). The post hoc power analysis for each group based on our actual sample sizes has been performed, assuming a two-sided significance level (α) of 0.05 and an effect size of 0.5 (medium effect size as per Cohen’s d). The power was calculated using the method from the statistical package in Python.

## Results

The demographic and clinical characteristics of the patients are presented in Table 1. Due to the limited data (n = 2) in the KL1 group, which could bias the analysis, this group was excluded. A post hoc power analysis was conducted. For KL2, KL3, and KL4 groups, the achieved powers were 41.0%, 54.1%, and 79.5%, respectively (significance level = 0.05). Ultimately, data from 53 patients (66 knees) were analyzed, including the distribution of patients and affected knees across KL2, KL3, and KL4 groups. The analysis covered age, sex, height, weight, BMI, occupation, and pre-injection knee range of motion. Data are presented as medians with ranges (minimum and maximum values).

**Table 1:** Demographic Data Continuous variables are reported as median (range). Categorical variables are expressed as absolute counts. KL: Kellgren-Lawrence.

Variable	KL2 Group	KL3 Group	KL4 Group
Number of patients (affected limbs)	12 (14)	16 (19)	25 (33)
Age (years)	60.5 (31 to 80)	68 (56 to 84)	70 (51 to 85)
Height (cm)	160.25 (143 to 181)	157.8 (140 to 167)	158 (147 to 177)
Weight (kg)	63.5 (43 to 82)	60 (42.5 to 75)	63 (49 to 120)
Body mass index (BMI)	23.21 (21.03 to 31.25)	26.48 (18.75 to 30.61)	24.32 (20.34 to 43.55)
Sex ratio (male/female)	6/6	3/13	9/16
Employment status (working/non-working)	9/3	12/4	8/17
Pre-injection maximum extension angle (°)	0 (-10 to 0)	-5 (-15 to 0)	-3 (-20 to 0)
Pre-injection maximum flexion angle (°)	145 (135 to 155)	140 (100 to 145)	140 (100 to 155)

Regarding KOOS scores, all groups of KOA patients showed improvements in scores one year after treatment compared to baseline. The MCID for KOOS scores in this study was as follows: pain, 7.6; symptoms, 8.4; activity, 7.8; sport, 12.0; and quality of life, 9.8. The number of patients exceeding the MCID of KOOS is shown in Table 2, and the responder rates were 78.6% in KL2, 68.4% in KL3, and 51.5% in KL4 (Table 3). Significant differences were observed in KOOS-S, KOOS-P, and KOOS-Q within all three groups (P < 0.05, Table 4). For KOOS-A and KOOS-SP, significant differences were only found in the KL2 and KL4 groups when comparing pre- and post-treatment scores (P < 0.05, Table 4). Additionally, significant differences were observed in pre- to post-treatment changes of KOOS-A and KOOS-Q scores across KL grade groups (P < 0.05; Tables 5, 6). Patients with mild KOA showed better recovery in activity and quality of life compared to those with moderate to severe KOA. Despite a trend toward greater improvements in milder KOA, a substantial proportion of patients with KL grade 4 still achieved the MCID thresholds for KOOS-S (54.55%), KOOS-A (57.58%), and KOOS-Q (45.45%), indicating meaningful clinical benefits even in more severe disease stages.

**Table 2:** KOOS Improvement in Patients with Different KL Grades (n, %) If the change in KOOS scores after one year of treatment (ΔKOOS) is ≥ MCID, it indicates that the treatment has achieved clinically meaningful improvement in the patient’s knee function or symptoms. Conversely, if ΔKOOS < MCID, it suggests that the improvement may not be sufficient for the patient to perceive a significant functional enhancement. "n" represents the number of patients with scores above MCID. KL: Kellgren-Lawrence, KOOS: Knee Injury and Osteoarthritis Outcome Score, KOOS-S: KOOS symptoms, KOOS-P: KOOS pain, KOOS-A: KOOS activity, KOOS-SP: KOOS sports, KOOS-Q: KOOS quality of life, MCID: Minimal Clinically Important Difference.

	KL2	KL3	KL4
KOOS-S	10 (71.43%)	10 (52.63%)	18 (54.55%)
KOOS-P	13 (92.86%)	15 (78.95%)	19 (57.58%)
KOOS-A	9 (64.29%)	6 (31.58%)	17 (51.52%)
KOOS-SP	9 (64.29%)	8 (42.11%)	15 (45.45%)
KOOS-Q	13 (92.86%)	13 (68.42%)	15 (45.45%)

**Table 3:** Responder and Non-responder Distribution With Responder Rates by KL Grade The responder rate is based on the number of knees. KL: Kellgren-Lawrence.

	KL2	KL3	KL4
Responder (n)	11	13	17
Non-responder (n)	3	6	16
Responder rate (%)	78.57%	68.42%	51.52%

**Table 4:** KOOS Score Comparison Within Groups (Pre-treatment vs. One-Year Post-treatment) This table presents the results of the within-group paired Wilcoxon paired rank-sum test. It compares the KOOS scores before treatment and at one-year follow-up for each group individually. * means < 0.05. KL: Kellgren-Lawrence, KOOS: Knee Injury and Osteoarthritis Outcome Score, KOOS-S: KOOS symptoms, KOOS-P: KOOS pain, KOOS-A: KOOS activity, KOOS-SP: KOOS sports, KOOS-Q: KOOS quality of life.

	P-Value		
KOOS	KL2 Group	KL3 Group	KL4 Group
KOOS-S	0.003*	<0.001*	<0.001*
KOOS-P	<0.001*	0.005*	<0.001*
KOOS-A	<0.001*	0.139	<0.001*
KOOS-SP	0.002*	0.132	0.002*
KOOS-Q	<0.001*	0.007*	0.001*

**Table 5:** Comparison of KOOS Score Changes Across KL Grades (One-Year Minus Baseline) The median and interquartile range (IQR) were used for descriptive statistics. The P-values resulting from the Kruskal-Wallis test conducted on the differences in KOOS scores (pre-treatment vs. one-year post-treatment) across the groups. Any statistically significant differences would be highlighted. * means < 0.05. KL: Kellgren-Lawrence, KOOS: Knee Injury and Osteoarthritis Outcome Score, KOOS-S: KOOS symptoms, KOOS-P: KOOS pain, KOOS-A: KOOS activity, KOOS-SP: KOOS sports, KOOS-Q: KOOS quality of life.

KOOS	KL2 Group	KL3 Group	KL4 Group	H Statistic	P-Value
KOOS-S	19.64 (3.57 to 28.57)	10.71 (1.79 to 19.64)	10.71 (0.00 to 14.29)	1.8957	0.388
KOOS-P	22.22 (14.58 to 29.86)	16.67 (8.33 to 22.22)	11.11 (0.00 to 22.22)	5.8962	0.052
KOOS-A	11.76 (4.78 to 20.59)	1.47 (0.00 to 8.82)	10.29 (0.00 to 20.59)	6.0261	0.049*
KOOS-SP	15.00 (10.00 to 23.75)	10.00 (0.00 to 20.00)	10.00 (-10.00 to 25.00)	1.6535	0.438
KOOS-Q	25.00 (18.75 to 35.94)	12.50 (3.12 to 25.00)	6.25 (0.00 to 25.00)	7.3361	0.026*

**Table 6:** Post Hoc Comparisons of KOOS and MOAKS Among the five KOOS subdomains, only KOOS-A and KOOS-Q showed significant one-year changes across KL grades; so only these were included in post hoc analysis. Among all MOAKS features, only inferior patellar osteophytes showed significant one-year changes across KL grades, so only this was included in post hoc analysis. Post hoc analyses with Bonferroni correction were applied for parameters showing statistically significant differences. Any statistically significant differences would be highlighted. * means < 0.05. KL: Kellgren-Lawrence, KOOS: Knee Injury and Osteoarthritis Outcome Score, KOOS-A: KOOS activity, KOOS-Q: KOOS quality of life, MOAKS: Magnetic Resonance Imaging Osteoarthritis Knee Score.

		P-Value		
Variables		KL2 vs. KL3	KL2 vs. KL4	KL3 vs. KL4
KOOS-A		0.007*	0.364	0.123
KOOS-Q		0.007*	0.008*	0.491
Osteophytes	Inferior Patella	0.005*	0.365	0.054

MOAKS-based imaging assessments revealed no significant differences in cartilage damage, BMLs, synovitis-effusion, and meniscal morphology and extrusion across KL grades before and after APS treatment, except for osteophytes. In the KL4 group, statistically significant progression was observed in both superior and inferior patellar osteophyte scores at one-year follow-up (P = 0.039, Table 7), with notably higher scores compared to baseline. Additionally, changes in inferior patellar osteophyte scores differed significantly among KL grades (KL2 vs. KL3 vs. KL4, P = 0.044, Table 8). These significant findings are visually illustrated in Appendix C.

**Table 7:** MOAKS Comparison Within Groups (Pre-treatment vs. One-Year Post-treatment) This table presents the results of the within-group paired Wilcoxon paired rank-sum test. It compares the MOAKS scores before treatment and at one-year follow-up for each group individually. If a "−" appears in the table, it indicates that there was no change between treatment before and after (i.e., the differences are all 0 for each data point). * means < 0.05. MOAKS: Magnetic Resonance Imaging Osteoarthritis Knee Score, KL: Kellgren-Lawrence.

	P-Value		
Variables	KL2 Group	KL3 Group	KL4 Group
Inferior patella osteophytes	>0.99	>0.99	0.039*
Superior patella osteophytes	0.063	-	0.039*

**Table 8:** Comparison of MOAKS Score Changes Across KL Grades (One-Year Minus Baseline) The median and interquartile range (IQR) were used for descriptive statistics. The P-values resulting from the Kruskal-Wallis test conducted on the differences in MOAKS scores (pre-treatment vs. one-year post-treatment) across the groups. Any statistically significant differences would be highlighted. * means < 0.05. MOAKS, Magnetic Resonance Imaging Osteoarthritis Knee Score; KL: Kellgren-Lawrence.

Variables	KL2 Group	KL3 Group	KL4 Group	H Statistic	P-Value
Inferior patella osteophytes	0.00 (0.00-1.00)	0.00 (0.00-0.00)	0.00 (0.00-0.00)	6.266	0.044*

## Discussion

This study systematically analyzed the therapeutic effects of APS in patients with varying severities of KOA using clinical scores and imaging assessments. The main findings are as follows: (1) While patients with milder KL grades demonstrated better overall responses, the MCID achievement rates in KL grade 4 patients highlight that APS can provide clinically meaningful improvements even in advanced KOA; (2) While APS treatment alleviated clinical symptoms in severe KOA patients, it did not significantly slow disease progression.

APS creates a treatment environment rich in anti-inflammatory molecules and growth factors through its high levels of anti-inflammatory cytokines and bioactive factors [7]. Previous studies have demonstrated that within six months of APS injection, patients showed significant improvements in Western Ontario and McMaster Universities Osteoarthritis Index (WOMAC) composite scores and subscales for pain, stiffness, and function. In some of these patients, the therapeutic effect lasted for more than 18 months [13]. The underlying mechanism may be related to the anti-inflammatory effect of APS, which alleviates inflammation and reduces pain in the short term, while its long-term efficacy may stem from the potential disease-relieving properties of joint homeostasis and cartilage quality [7,13]. In addition, research by Hix et al. also reported significant improvements in KOOS scores for symptoms, stiffness, function, and sports across follow-up time points, further supporting APS's potential in symptom relief and joint function improvement [20]. In this study, we observed similar findings: KOOS scores improved at 12 months post-treatment across all KOA severity levels, with more pronounced improvements in mild KOA. This can be attributed to the relatively intact cartilage and joint homeostasis in mild KOA, allowing the anti-inflammatory and growth factors in APS to work more effectively in suppressing inflammation, protecting cartilage, and promoting repair [10]. In addition, inflammation serves as a key feature associated with joint symptoms and disease progression in OA [21]. APS provides rapid symptomatic relief, which makes it more effective in early treatment. However, the observed differences in KOOS improvement patterns between KL2, KL3, and KL4 groups may reflect underlying variations in disease status. KL3 patients often exhibit a transitional stage of joint degeneration, with more advanced cartilage and subchondral damage than KL2, but without the extensive structural destruction typical of KL4. This heterogeneity in joint pathology may contribute to more variable clinical responses to APS, resulting in different improvement patterns compared to the other groups. There are also some concerns about the treatment of APS. The high concentration of leukocytes in APS has raised concerns, as some researchers suggest that leukocyte-rich PRP may exert catabolic effects through the release of catabolic proteins or pro-inflammatory molecules, potentially impacting therapeutic outcomes [22]. Additionally, some studies have reported that after initial improvements, APS’s efficacy may not differ significantly from the blank control group over time [23,24]. Further research is needed to explore the potential mechanisms of leukocytes in APS's clinical effects on OA. Nevertheless, the clinical outcomes observed in this study provide strong support for APS as an effective treatment in improving OA symptoms.

Currently, most of the clinical observational studies on APS mainly use the scoring system as the main index to assess the efficacy. In contrast, a major feature of this study is the systematic and detailed analysis of various subregions of knee MRI images by MOAKS scoring. Improvement of cartilage damage has been controversial as a key target of observation in APBO therapy. Previous researches have shown that PRP may not significantly improve cartilage structural damage [25]. Similarly, in APS-related studies, Kon et al. observed APS injections for KOA at one-year and three-year follow-ups and did not find any significant changes in cartilage [10,11]. The results of the present study are consistent with these studies in that no significant therapeutic effect of APS on cartilage damage was observed after 12 months of treatment. However, in vitro studies have found that APS contains a variety of anti-inflammatory cytokines, including IL-1 receptor antagonists and soluble TNF-α receptors I and II, which exert a protective effect on cartilage by antagonizing IL-1β and TNF-α to inhibit the synthesis of matrix metalloproteinases (MMPs) [7]. In addition, the anabolic factors in APS (e.g., epidermal growth factor (EGF), insulin-like growth factor-1 (IGF-1), PDGF-AB/-BB, and VEGF, etc.) were significantly increased in APS, which also contributed to the proliferation of chondrocytes [26]. Matuska et al. further validated the ability of APS to protect cartilage and promote chondrocyte proliferation through a study based on bovine cartilage explants [27]. They proposed that anti-inflammatory factors in APS may be critical in driving the chondroprotective effects, while anabolic growth factors may contribute significantly to chondrocyte proliferation [27]. Nevertheless, these theoretical mechanisms have not demonstrated significant effects in clinical observations. This result may be attributed to the difficulty of in vitro experiments to fully reflect the complex pathophysiologic environment and the interactions between multiple cell populations in the joint cavity. Such differences in results may also be attributed to various other factors. First, a single intra-articular injection may not deliver a sufficient therapeutic dose to sustain cartilage protection or promote structural repair. Second, reliance on imaging assessments at a single time point may limit the ability to detect progressive or delayed structural changes. Therefore, more clinical observations and mechanism studies are needed to further clarify whether APS can produce significant effects on cartilage damage in the treatment of knee osteoarthritis and to develop a consistent scientific consensus.

In the KL classification of KOA, bone capillary formation, joint space narrowing, osteophyte formation, and joint deformity are the key indicators for evaluating disease progression and severity. Therefore, the changes of the osteophyte reflect the progression and severity of the disease to a certain extent [28]. In this study, we found that osteophytes at the superior and inferior poles of the patella continued to progress after 12 months of APS treatment in severe KOA. This suggests that in severe KOA, APS treatment did not significantly improve the disease process and failed to effectively slow down the progression of the disease. Analyzing the reasons, the progression of patellar osteophytes may be closely related to abnormal mechanical stress, chronic inflammatory stimulation, and cartilage damage [29,30]. As a high-stress area of the patellofemoral joint, the superior and inferior poles of the patella further concentrate the mechanical load after cartilage degeneration; imbalances of quadriceps function and joint deformity exacerbate the uneven distribution of stress [31]. Additionally, synovitis and pro-inflammatory factors in joint fluid (e.g., IL-1β and TNF-α) stimulate overexpression of osteogenic factors (e.g., TGF-β and bone morphogenetic proteins (BMPs)), causing excessive subchondral bone reactions and accelerating osteophyte formation [29]. Furthermore, the chronic shear and friction forces exerted on the superior and inferior poles of the patella worsen osteophyte progression [30]. Despite the strong anti-inflammatory effects of APS, the findings suggest limitations in its efficacy, failing to achieve the desired results in severe KOA. This finding provides an important reference for the selection of the applicable population for APS treatment.

The progression of KOA is closely associated with joint effusion, synovitis, BMLs, and meniscal lesions [32]. Therefore, on MRI images, changes in these areas are equally responsive to treatment outcomes. Although in this study, no significant differences in the above lesions were observed before and after treatment and between patients with different KL grading. However, prior studies have demonstrated that APS and PRP applications can improve BMLs and joint effusion in OA patients [11]. It is hypothesized that this difference may be related to the heterogeneity of the patient population or variability in the treatment process, but this hypothesis still needs to be verified by more relevant clinical studies and observations to further clarify its general pattern and clinical significance. Due to the relatively small sample size of this retrospective study, certain structural improvements may not have been detected. Therefore, the absence of significant changes in MRI structural outcomes based on MOAKS highlights the need for larger-scale cohort studies in the future to further validate these findings.

This study has some limitations: first, as a retrospective study, this study only reviewed the clinical outcomes of APS treatment in the uncontrolled group, and there was no control group, which did not allow for a clear assessment of the superiority of APS treatment compared to other treatment modalities (or a blank control). Moreover, the absence of a control group makes it difficult to distinguish the true effect of APS from the natural course of the disease, placebo effect, or regression to the mean. These confounding factors may lead to an overestimation of the observed treatment benefits. Therefore, prospective controlled studies are warranted to validate the true efficacy of APS. Second, this study only evaluated outcomes at the starting and end points of treatment and failed to understand trends in changes throughout treatment. In addition, this study only assessed the therapeutic effect of a single injection of APS, and the potential effect of injection frequency on efficacy has not been clarified. Third, the relatively small sample size and the absence of a formal statistical power analysis may limit the robustness and generalizability of our conclusions. While we observed significant improvements in patient-reported outcomes, it is important to recognize that these subjective gains may be driven in part by placebo responses or by the short-term anti-inflammatory milieu provided by APS, rather than by true cartilage regeneration. Osteoarthritis is typically a slowly progressive disease, with structural deterioration occurring over multiple years; thus, the absence of detectable MRI changes at one year aligns with the expected natural course of KOA. Given our retrospective, uncontrolled design, this study can be considered as hypothetical research. Definitive conclusions regarding APS’s ability to modify disease progression will require prospective, randomized, placebo- or active-controlled trials with longer follow-up and formal imaging endpoints.

## Conclusions

This study evaluated the therapeutic efficacy of a single injection of APS in patients with different KL grades. The results showed that KOOS scores improved 12 months after treatment, but the improvement may be greater in mild KOA than in severe KOA. However, APS did not produce a significant improvement in structural lesions as assessed by MOAKS imaging over one year. These findings may suggest that APS has potential clinical application in the treatment of KOA, especially in patients with mild disease, although further prospective controlled studies are needed to confirm these preliminary observations.

## Figures and Tables

**Figure 1:**
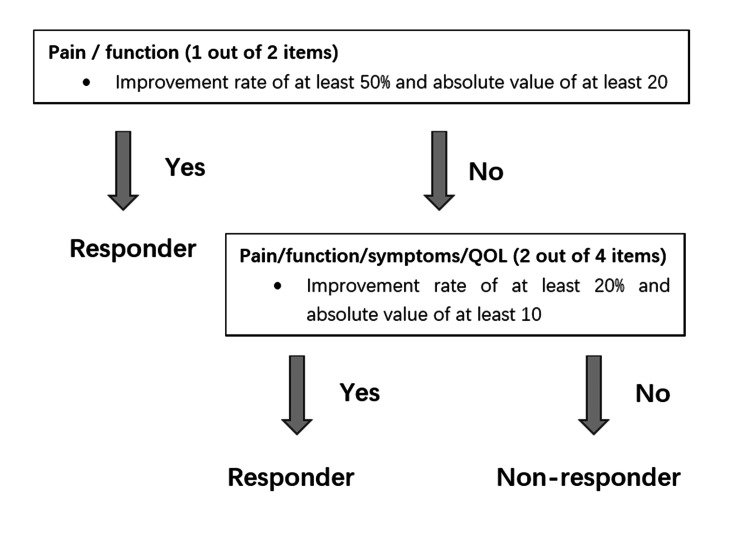
The Outcome Measures in Arthritis Clinical Trials-Osteoarthritis Research Society International (OMERACT-OARSI) Criteria QOL: quality of life.

**Figure 2:**
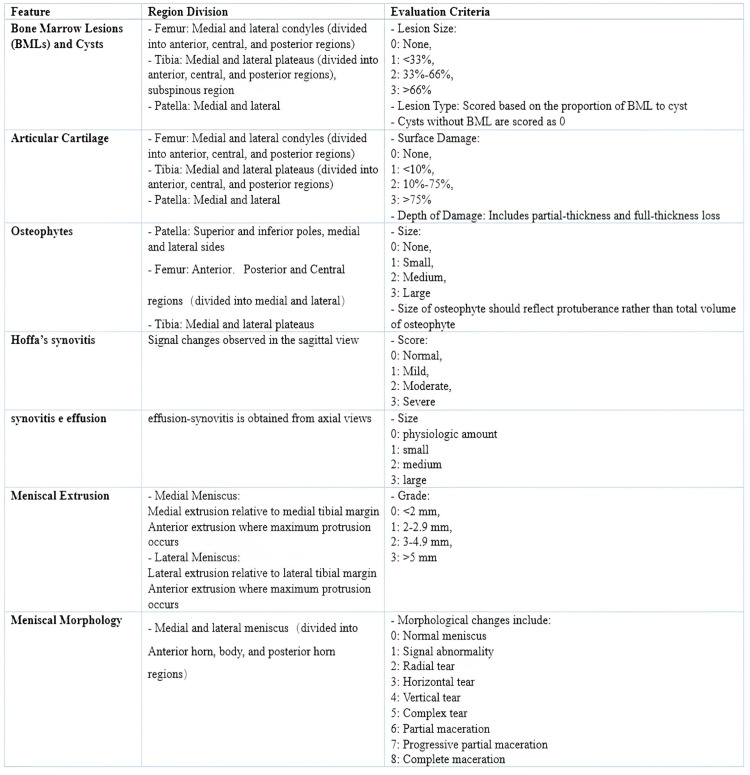
MRI Osteoarthritis Knee Score (MOAKS) Feature

**Figure 3:**
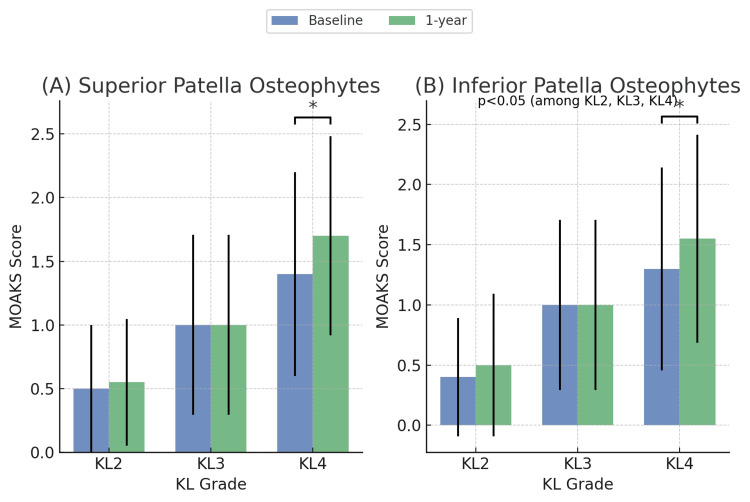
Changes in MRI Osteoarthritis Knee Score (MOAKS) by KL Grade Group (Baseline vs. One-Year Follow-Up) (A) Superior patella osteophytes. (B) Inferior patella osteophytes. * indicates that, in the KL4 group, the baseline vs. one-year comparison yielded P < 0.05. KL: Kellgren-Lawrence.

## Appendices

Appendix A 

Appendix B 

Appendix C 
